# Iterative reconstruction incorporating positron range correction within STIR framework

**DOI:** 10.1186/2197-7364-1-S1-A42

**Published:** 2014-07-29

**Authors:** Ottavia Bertolli, Matteo Cecchetti, Niccolò Camarlinghi, Afroditi Eleftheriou, Nicola Belcari, Charalampos Tsoumpas

**Affiliations:** Division of Medical Physics, University of Leeds, Kragujevac, UK; Department of Physics, University of Pisa and INFN Pisa, Kragujevac, Italy; Department of Physics, National and Kapodistrian University of Athens, Kragujevac, Greece

Positron range is one important physical limitation to spatial resolution in PET imaging. We present a method to take account of positron range blurring in iterative reconstruction by including specific positron range kernels approximating the annihilation distribution in the forward projection operation [1]. The correction method is implemented within the OSMAPOSL iterative reconstruction algorithm of STIR 3.0 [2].

GATE [3] simulation package was used to obtain the positron annihilation distribution of a point source of 68Ga placed in the centre of spherical water and lung tissue phantoms. The simulations were performed without and with 3T static magnetic field in the axial direction. To obtain the kernels the annihilation coordinates were voxelized into a 3D matrix with element size equal to the reconstructed image's voxel size and then normalised. The proposed method was evaluated using simulation data (generated with STIR) of a parallelepiped water phantom containing two spherical hot spots, on the HYPERImage preclinical PET/MR scanner [4]. To simulate the positron range effect the phantom was blurred by convolution with the positron kernel relative to water. The blurred images were reconstructed with and without filtering of the forward projected image.

Line profiles traced through the spots of the reconstructed images show that the use of the positron range correction yields sharper boundary definition, albeit with noise enhancement. The values relative to the spots volume show that the correction results in the recovery of the activity mean value compared to the simulated phantom (e.g. 55% to 94% for the smaller spot, no magnetic field).

Furthermore the tissue-dependent correction, which consists in blurring the image with the kernel correspondent to the voxel tissue (water or lung), was implemented and evaluated on a phantom with water and lung regions. The activity mean value is recovered but the method performance in correspondence to the edges needs further investigation.
